# AFM and FTIR Investigation of the Effect of Water Flow on Horseradish Peroxidase

**DOI:** 10.3390/molecules26020306

**Published:** 2021-01-09

**Authors:** Yuri D. Ivanov, Tatyana O. Pleshakova, Ivan D. Shumov, Andrey F. Kozlov, Anastasia A. Valueva, Irina A. Ivanova, Maria O. Ershova, Dmitry I. Larionov, Victor V. Repnikov, Nina D. Ivanova, Vadim Yu. Tatur, Igor N. Stepanov, Vadim S. Ziborov

**Affiliations:** 1 Institute of Biomedical Chemistry, Moscow 119121, Russia; t.pleshakova1@gmail.com (T.O.P.); shum230988@mail.ru (I.D.S.); afkozlow@mail.ru (A.F.K.); varuevavarueva@gmail.com (A.A.V.); i.a.ivanova@bk.ru (I.A.I.); motya00121997@mail.ru (M.O.E.); corvus.coraxnm@gmail.com (D.I.L.); ziborov.vs@yandex.ru (V.S.Z.); 2 Bruker Ltd., Moscow 119017, Russia; viktor.repnikov@bruker.com; 3 Skryabin Moscow State Academy of Veterinary Medicine and Biotechnology, Moscow 109472, Russia; ninaivan1972@gmail.com; 4 Foundation of Perspective Technologies and Novations, Moscow 115682, Russia; v_tatur@mail.ru (V.Y.T.); fptn@mail.ru (I.N.S.); 5 Joint Institute for High Temperatures of the Russian Academy of Sciences, Moscow 125412, Russia

**Keywords:** horseradish peroxidase, protein adsorption, protein aggregation, electromagnetic field, triboelectric effect

## Abstract

Atomic force microscopy (AFM)-based fishing is a promising method for the detection of low-abundant proteins. This method is based on the capturing of the target proteins from the analyzed solution onto a solid substrate, with subsequent counting of the captured protein molecules on the substrate surface by AFM. Protein adsorption onto the substrate surface represents one of the key factors determining the capturing efficiency. Accordingly, studying the factors influencing the protein adsorbability onto the substrate surface represents an actual direction in biomedical research. Herein, the influence of water motion in a flow-based system on the protein adsorbability and on its enzymatic activity has been studied with an example of horseradish peroxidase (HRP) enzyme by AFM, attenuated total reflection Fourier-transform infrared spectroscopy (ATR-FTIR) and conventional spectrophotometry. In the experiments, HRP solution was incubated in a setup modeling the flow section of a biosensor communication. The measuring cell with the protein solution was placed near a coiled silicone pipe, through which water was pumped. The adsorbability of the protein onto the surface of the mica substrate has been studied by AFM. It has been demonstrated that incubation of the HRP solution near the coiled silicone pipe with flowing water leads to an increase in its adsorbability onto mica. This is accompanied by a change in the enzyme’s secondary structure, as has been revealed by ATR-FTIR. At the same time, its enzymatic activity remains unchanged. The results reported herein can be useful in the development of models describing the influence of liquid flow on the properties of enzymes and other proteins. The latter is particularly important for the development of biosensors for biomedical applications—particularly for serological analysis, which is intended for the early diagnosis of various types of cancer and infectious diseases. Our results should also be taken into account in studies of the effects of protein aggregation on hemodynamics, which plays a key role in human body functioning.

## 1. Introduction

Early diagnosis of oncological diseases in humans requires the detection of cancer-associated marker proteins at femtomolar (10^−15^ M) or, better, even lower concentrations [1]. This is why the development of novel highly sensitive bioanalytical systems, which are able to overcome the 10^−15^ M threshold, is an acute problem of modern biomedical research. Such highly sensitive systems are based on the so-called molecular detectors, capable of registering single target molecules [2,3]. In the majority of analytical systems, intended for the highly sensitive detection of proteins, a principle of molecular fishing is realized [3]. This principle is based on the capturing of the target biomolecules from the volume of a liquid sample onto the surface of a solid substrate, and the binding of these molecules with the substrate is registered with a detector. In particular, atomic force microscopy (AFM)-based molecular fishing allows one to detect proteins at ultra-low (down to 10^−17^ M) concentrations [4]. The practical implementation of the AFM-based fishing approach comprises two main steps: (1) the incubation of the AFM substrate (the so-called AFM chip, onto which the target biomolecules are to be captured [4,5]) in the analyzed sample; and (2) counting of the captured target molecules on the substrate surface with an atomic force microscope. In [3], it was emphasized that reliable realization of the first step requires an efficient delivery of the target biomolecules from the sample solution onto the substrate surface. For this purpose, hydrodynamic intensification (that is, intensive stirring of the analyzed sample) of the delivery process is often used [3,4,6]. Nevertheless, despite the importance of the intensive delivery of the target biomolecules to the substrate surface, the overall possibility of their capturing is governed by their adsorbability onto this surface. The latter is determined by electrostatic and hydrophobic interactions between the biomolecules and the substrate surface. This is why the adsorption properties of proteins should be investigated.

In the development of bioanalytical systems, the following factor should also be considered. Since most proteins are heat-sensitive, thermal stabilization of measurement cells is required in such systems. This is often realized by employing flow-based heat exchangers [7], such as coils, where water is used as a heat-transfer agent. The flow of water through a coiled polymeric pipe was shown to induce an electromagnetic field, which can influence protein molecules, causing changes in their physicochemical properties [8]. Such a field, induced by liquid flow owing to a triboelectric effect, can extend to outside the coil. For this reason, it is interesting to study the influence of the flow-induced electromagnetic field on protein samples, incubated near a coiled pipe outside the coil.

**Figure 1:**
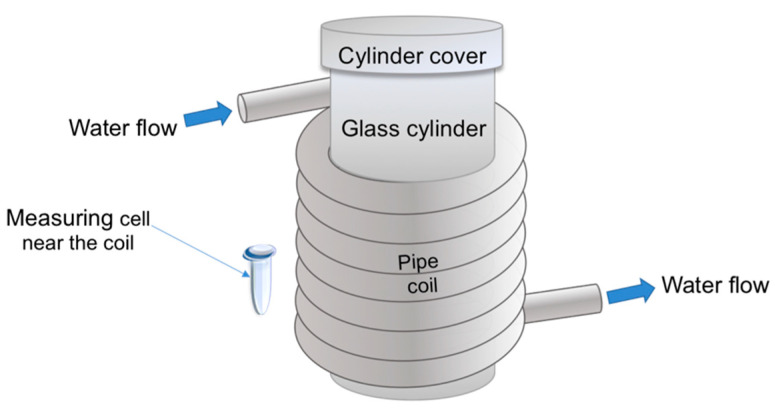
Schematic representation of the experimental setup employed for studying the effect of water flow on the properties of a protein. The measuring cell with the enzyme solution was placed either near the coil or far away. The silicone pipe is spiral-wound onto a glass cylinder to form a coil; water is pumped through the coiled pipe.

Accordingly, we have studied the influence of an electromagnetic field, induced by the flow of water through a coiled polymeric pipe, on the adsorption properties of a protein, whose aqueous solution was incubated near the pipe outside the coil. Such a layout (Figure 1) can be realized when the protein fishing is performed simultaneously in a number of measuring cells. In control experiments, the measuring cell with the sample protein solution was placed far away, at a 10 m distance from the coil.

Herein, horseradish peroxidase (HRP) has been employed as a model protein. Its properties are well known, and this is why it has been employed in our experiments. HRP, which pertains to heme-containing enzymes, represents a glycoprotein, whose molecular weight makes up about 40 kDa to 44 kDa [9,10]. HRP catalyzes the oxidation of a wide range of both organic and inorganic compounds by hydrogen peroxide [11]. The HRP structure includes 77% α-helices and 12% β-sheets [12]. The HRP macromolecule includes 18% to 27% of carbohydrate chains, which stabilize the protein structure [10,13,14].

The adsorbability of HRP has been investigated by AFM using the direct surface adsorption method [15] of HRP from the sample solutions onto mica substrates, which are commonly employed in AFM studies [16]. AFM allows one to perform visualization of individual enzyme molecules [17]; this is of interest for single-molecule enzymology.

The physicochemical properties of HRP have been additionally studied by attenuated total reflection Fourier-transform infrared spectroscopy (ATR-FTIR). FTIR is commonly employed to study protein secondary structures [18,19,20] and protein–protein interactions [21,22]. This method represents a useful tool in monitoring changes in protein secondary structure within the range of Amide I and Amide II bands, from 1700 to 1500 cm^−1^ [23]. ATR-FTIR finds its application in studying proteins in insoluble and aggregated states [24].

In parallel, the enzymatic activity of HRP has been monitored by conventional spectrophotometry. In our research, these three methods have been employed together to study the influence of a flow-induced electromagnetic field on the properties of the HRP enzyme protein.

Herein, we used a spiral-wound (coiled) silicone pipe modeling a biosensor’s flow section (see Figure 1). A standard Eppendorf-type polypropylene test tube, modeling a biosensor’s measuring cell, placed near and outside the coil, contained the test solution of the HRP model protein (Figure 1). By AFM, an increased adsorbability of the HRP protein structures has been revealed after the incubation of the HRP solution near the coil with flowing water. Moreover, a change in the mutual intensity of Amide II to Amide I has been observed in the ATR-FTIR spectra. This indicates a change in the HRP secondary structure, occurring during its incubation near the coil. At that, no change in the enzymatic activity of HRP has been observed.

The results obtained herein can be useful in biosensor-based studies of the structure of proteins and their complexes. Our results can also be used in the development of serological methods for the early diagnosis of diseases (such as brain cancer, prostate cancer, etc.) in humans. These data can also be of use in studying hemodynamics in the human body.

## 2. Results

### 2.1. Atomic Force Microscopy Visualization of Mica-Adsorbed HRP

**Figure 2:**
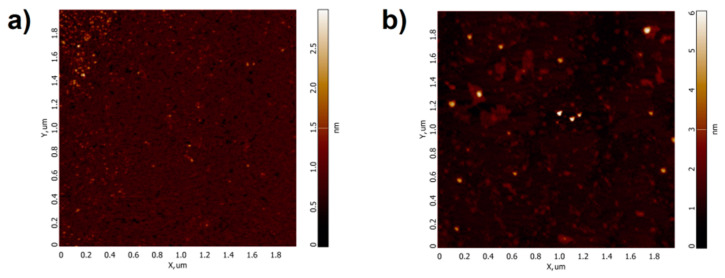
Typical atomic force microscopy (AFM) images of the surface of mica substrate with horseradish peroxidase (HRP) biomolecules adsorbed from the protein solution, which was incubated either far away at a 10 m distance from the polymeric pipe coil (control experiment, (**a**)), or near the coil (**b**).

**Figure 3:**
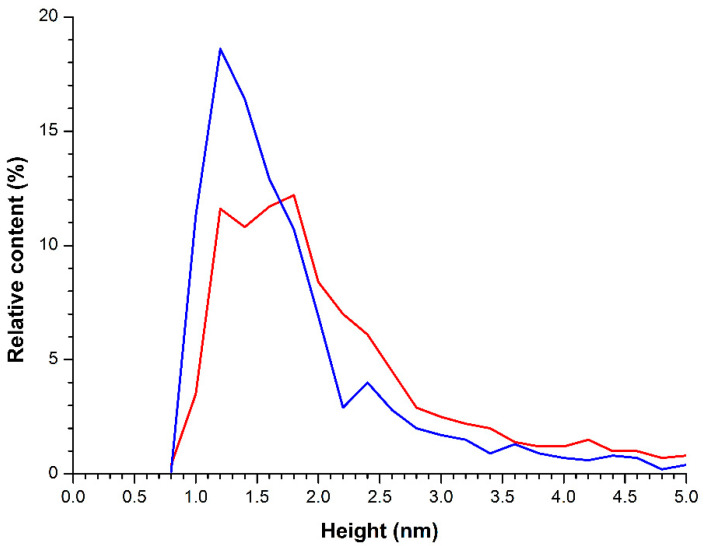
Results of processing of the AFM data obtained upon the analysis of HRP solutions. Typical plots of the relative distribution of the imaged objects with height *ρ(h).* The cell with HRP solution was placed either far away at a 10 m distance from the polymeric pipe coil (control experiment, blue line), or near the coil (red line).

Figure 2 displays typical AFM images of HRP biomolecules, adsorbed onto mica substrates from the working solutions (incubated near the coil) and from the control solution (incubated far away from the coil). Figure 3 displays the plots of distributions of the visualized objects with height *ρ(h)*, obtained after processing AFM data, which were summarized for each sample.

The images shown in Figure 2a indicate that in the control experiment, when the HRP solution was incubated far away from the coil, the protein adsorbs onto the substrate surface in the form of compact objects, whose heights are in the range of 1.0 nm to 4.0 nm. The corresponding *ρ(h)* plot (Figure 3, blue line) indicates that the maximum number of the mica-adsorbed molecules is of (1.2 ± 0.2) nm in height, and can thus be attributed to monomeric HRP. Indeed, these data are in agreement with the height values (1.6 ± 0.1 nm) obtained for AFM-visualized HRP captured onto chemically activated amino mica from its ultra-low-concentration (10^−17^ M) aqueous solution [4]. Moreover, the molecular weight *M_r_* of HRP is known to be 40 to 44 kDa [9,10], while other proteins with similar molecular weight—such as putidaredoxin reductase (*h_max_* = 1.8 nm [25], *M_r_* = 45.6 kDa [26]) and adrenodoxin reductase (*h_max_* = 1.8 nm [27], *M_r_* = 54 kDa [28])—were also reported to have comparable sizes.

At the same time, the *ρ(h)* plot, obtained for the control HRP solution (blue solid line in Figure 3), has an inflection point near 2.2 nm—that is, the *ρ(h)* curve is characterized by a non-monotonic dip near this point. This indicates an additional contribution from higher objects (i.e., from aggregated HRP) to the right wing of the *ρ(h)* distribution, corresponding to the heights >2.2 nm. That is, HRP was observed to adsorb from the control solution onto mica substrates in the form of a mixture containing both monomeric and aggregated enzyme.

Regarding the case with the HRP solution incubated near the coil with flowing water (Figure 2b), HRP biomolecules adsorb onto mica in the form of compact objects, whose heights range from 1.0 nm to 4.0 nm—similar to the case with the control solution. The general behavior of the corresponding relative distribution curve *ρ(h)* (Figure 3, red solid line) is similar to that of the *ρ(h)* curve obtained for the control sample, but the curve itself is somewhat shifted to the right. This indicates that after the exposure of the HRP solution to the flow-induced electromagnetic field, the protein exhibits a tendency to adsorb onto mica in aggregate form.

**Figure 4:**
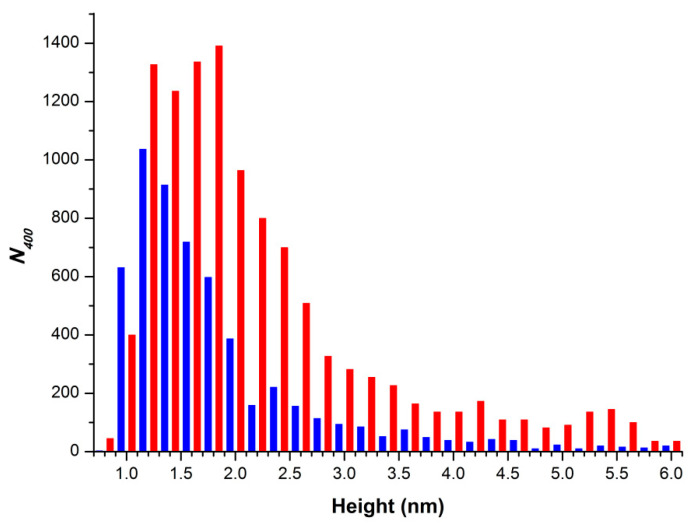
Results of processing of data obtained upon the AFM analysis of HRP solutions. Typical histograms displaying the absolute number of AFM-visualized objects, normalized per 400 μm^2^ area. The cell with HRP solution was placed either far away at a 10 m distance from the polymeric pipe coil (blue bars), or near the coil (red bars).

Figure 4 shows a typical histogram displaying the absolute number of AFM-visualized objects, normalized per 400 μm^2^ area, obtained for the HRP solutions, which were incubated either near the coil (Figure 4, red bars) or far away from the coil (Figure 4, blue bars).

The data shown in Figure 4 clearly indicate a 1.5-fold to twofold increase in the number of objects with heights >1.2 nm, adsorbed onto the mica surface from the HRP solution, incubated near the coil, in the entire range of heights—in comparison with the data obtained for the control solution. This fact indicates an increase in the adsorbability of HRP after the exposure of its solution to the flow-induced electromagnetic field.

### 2.2. ATR-FTIR Revelation of Changes in the HRP Secondary Structure

**Figure 5:**
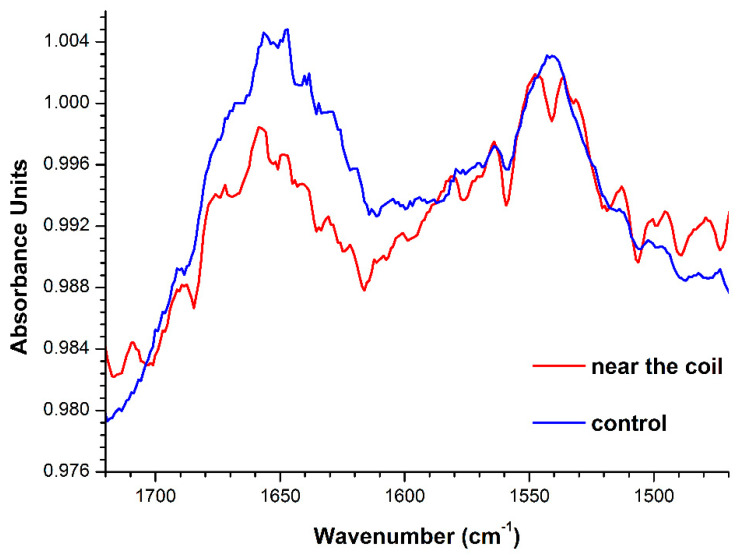
Attenuated total reflection Fourier-transform infrared spectroscopy (ATR-FTIR) spectra obtained for 10^−4^ M HRP solutions, which were incubated either near the coil with flowing water (red line) or at a 10 m distance from the coil (blue line).

Figure 5 displays the results of ATR-FTIR measurements of spectral characteristics of 10^−4^ M HRP solutions, which were incubated either near the coil with flowing water or far away from the coil.

As one can see from Figure 5, for the control HRP solution, incubated at a 10 m distance from the coil, two characteristic peaks at 1650 cm^−1^ (Amide I) and 1524 cm^−1^ (Amide II) are observed within the 1500 cm^−1^ to 1700 cm^−1^ wavelength range. A change in the ATR-FTIR spectrum was observed within this range for the HRP solution incubated near the coil. Namely, a change in the ratio between the intensities of the peaks, corresponding to Amide I and Amide II, was registered. These results indicate a decrease in the intensity near 1650 cm^−1^ for the protein solution incubated near the coil—in comparison with that obtained for the control solution. For the 1524 cm^−1^ band, no change in the intensity is observed. That is, the intensity of the Amide I peak decreases relatively to that of the Amide II peak.

Thus, with the example of HRP, an electromagnetic field, induced by the water flow through a polymeric pipe coil, has been found to affect the spectral characteristics of aqueous protein solutions.

### 2.3. Spectrophotometric Estimation of the HRP Enzymatic Activity

**Figure 6:**
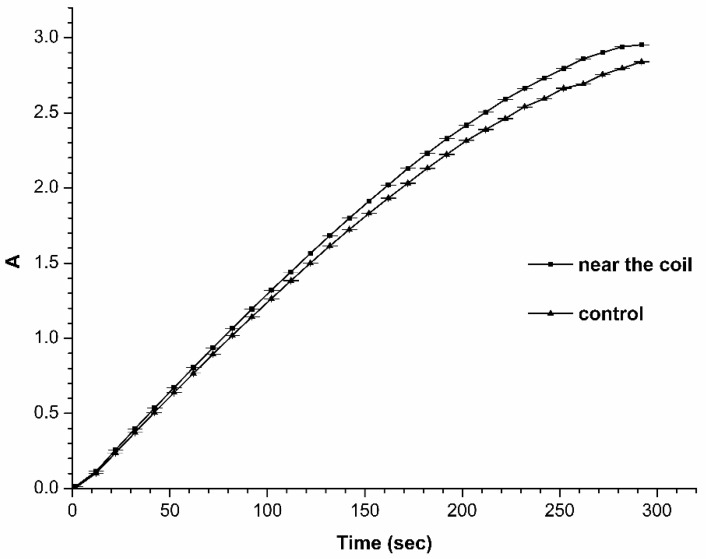
Spectrophotometric estimation of enzymatic activity of HRP. Characteristic time dependencies of change in solution absorbance at 405 nm obtained for the control HRP sample, incubated far away at a 10 m distance from the silicone pipe coil (squares), and for the HRP sample incubated near the coil (triangles). Experimental conditions: HRP:ABTS:H_2_O_2_ = 10^−9^ M:3 mM:2.5 mM. T = 23 °C.

The enzymatic activity of HRP has been estimated by monitoring the change in light absorbance (A_405_) of the HRP solution in the course of its reaction with the 2,2′-azino-bis(3-ethylbenzothiazoline-6-sulfonate) (ABTS) substrate, as described in Materials and Methods. Figure 6 displays the typical time dependencies of A_405_, obtained for the HRP solution incubated near the coil and for that incubated far away from the coil. The curves shown in Figure 6 indicate that the incubation of the HRP solution near the coil does not lead to any somewhat considerable change in the enzymatic activity of HRP—in comparison to the case with the control solution, incubated at a 10 m distance from the coil.

## 3. Discussion

In the present research, we have studied the influence of an electromagnetic field, induced by the water flow through a coiled polymeric pipe, on the adsorption properties of a model protein, whose solution was placed near the coil. By AFM, a 1.5- to 2-fold increase in the adsorbability of HRP onto mica has been revealed. At that, no change in its enzymatic activity was observed. This indicates a slight change in the enzyme structure, which does not affect the active site. Nevertheless, it should be emphasized that the strength of the inductive field, arising upon the motion of liquid, is sufficiently high, and this is indicated by changes in the spectral characteristics of the HRP solution within the 1500 to 1700 cm^−1^ range, observed in our ATR-FTIR experiments. Within the 1500 to 1700 cm^−1^ range, ATR-FTIR spectra reflect specific features of the protein secondary structure [23]. HRP glycoprotein is known to contain 77% ɑ-helices [12]. We have revealed that the electromagnetic field, induced by the flow of water through the polymeric coil, induces a shift in the Amide I to Amide II ratio towards the decrease in Amide I. The Amide I band (1700 to 1600 cm^−1^) corresponds to C=O stretching vibrations of peptide linkages at 1621 cm^−1^, while the Amide II band (1620 to 1500 cm^−1^) is caused by a combination of N-H deformational vibrations and C-N stretching vibrations of the peptide groups [23]. That is, changes in the HRP secondary structure are clearly observed—but in such a way that the structure of the active site is not affected. Accordingly, one can conclude that the flow-induced electromagnetic field causes changes in the HRP structure near the surface of the protein globule, while not affecting the active site.

The observed effect of the water, flowing through the coiled silicone pipe, on the adsorbability of HRP is apparently explained by the electromagnetic nature of the flow-induced field. The induction of such an electromagnetic field obviously occurs owing to a triboelectric effect consisting of a generation of charge in the liquid, flowing along a polymeric surface [8,29,30,31,32,33,34]. The triboelectrically induced charges induce an electromagnetic field, which, in turn, affects the protein during the incubation of its solution near the coil. It is to be emphasized that the enzymatic activity of the model protein, estimated by spectrophotometry, remains virtually unchanged, despite the change in its adsorbability onto mica observed by AFM. This fact indicates that the flow-induced field only impacts the spatial structures of the protein globule near its surface, while not affecting its active site. The effects of HRP aggregation in the presence of electromagnetic fields were previously reported in our recent studies [8,35,36] and by Sun et al. [37,38].

The results reported herein indicate the effect of an electromagnetic field, induced by water flow through a polymeric pipe coil, on a solution of HRP enzyme incubated near this coil. It should be emphasized that HRP has just been used as a convenient model object which can form aggregates—similar to many other enzymes. The approach proposed herein can be well used for studying liquid flow-induced effects on other proteins, which play important functional roles in the functioning of the human body and other living systems. The functional activity of many proteins (including enzymes) is known to be dependent on their aggregation state. Cytochrome P450 BM3 represents one of the examples of such proteins. This protein pertains to the superfamily of heme-containing cytochromes P450. It represents a 119-kDa water-soluble enzyme [39], comprising two domains: a reductase one and a heme-containing one [40]. BM3 catalyzes fatty acids monooxygenation in bacteria [39]. The full-length BM3 protein can exist in monomeric and oligomeric forms, but is mainly (>50%) present in dimeric form [40,41]. The activity of the dimeric form of cytochrome P450 BM3 was shown to be an order of magnitude higher than that of its monomeric form [42].

Myeloperoxidase, participating in atherogenesis in humans, represents another example of an enzyme which is functionally active in dimeric form [43].

Regarding non-enzymatic proteins, FXR1 is a typical example of a non-enzymatic protein which is functionally active in amyloid form, representing stacked monomers stabilized by intermolecular β-sheets. This protein regulates memory and emotions [44,45] and is assumed to be present in amyloid form in the brains of various mammals, including humans [46].

Our results should be taken into account in the formulation of models in which the physicochemical properties of proteins are considered—for instance, in modeling pathological processes involving enzymes in the form of functional multiprotein complexes (such as inflammatory processes occurring with the participation of myeloperoxidase [43]). The phenomenon observed is also to be taken into account in the development of biosensor systems. This is particularly important in highly sensitive biosensors, intended for the diagnosis of various types of cancer (such as brain cancer, prostate cancer, breast cancer, and ovarian cancer) in humans [35]. Since the peroxidase enzyme changes its adsorbability under the influence of an electromagnetic field, then the influence of electromagnetic radiation on inflammatory processes is also possible.

## 4. Conclusions

Herein, with the example of studying the change in the aggregation state of the HRP protein by AFM, it has been shown that a water flow, formed in flow sections of biosensors, has an effect on an enzyme. It has been demonstrated that, after the exposure of the HRP solution to the electromagnetic field induced by the water flow, an increase in both the adsorbability of HRP and its aggregation state onto mica occurs. Moreover, the ATR-FTIR measurements have revealed changes in the secondary structure of the enzyme, while it retains its enzymatic activity. The results reported herein can be useful in the interpretation of data obtained during the protein studies involving biosensors with flow-based systems. The effect observed herein is also important to be taken into account in biomedical applications—particularly in the development of biosensors intended for serological assays for the early diagnosis of various types of oncological and infectious diseases. Our results should also be taken into account in studying the effects of protein aggregation on hemodynamics, which plays a key role in human body functioning.

## 5. Materials and Methods

### 5.1. Chemicals and Protein

Peroxidase from horseradish (HRP-C; Cat.# P6782) was obtained from Sigma (St. Louis, MO, USA). From Sigma, 2,2′-azino-bis(3-ethylbenzothiazoline-6-sulfonate) (ABTS) was purchased. Disodium hydrogen orthophosphate (Na_2_HPO_4_), citric acid and hydrogen peroxide (H_2_O_2_) were purchased from Reakhim (Moscow, Russia). All solutions were prepared using deionized ultrapure water (of 18.2 MΩ × cm resistivity) obtained with a Simplicity UV system (Millipore, Molsheim, France).

### 5.2. Experimental Setup

Experimental setup, which is analogous to the one used in our previous study [8], is schematically shown in Figure 1 (see the Introduction). The coil, through which water was pumped at a speed of 4 m/s, was represented by a silicone pipe (outer diameter 10 mm), spiral-wound (13 turns) onto a 170 mm diameter glass cylinder, so that the coil length was 130 mm.

A standard 1.7 mL Eppendorf-type polypropylene tube modeled a measuring cell, into which 1 mL of 0.1 μM (10^−7^ M) aqueous solution of HRP was placed. The cell was placed near the coil, i.e., aside from the coil at a 0.3 mm distance (Figure 1). The working experiments were performed at room temperature (23 °C). After that, the solution was subjected to AFM, ATR-FTIR and spectrophotometric analysis. In control experiments, the cell with HRP solution was placed far away (at a distance of 10 m) from the experimental setup for 40 min. After that, the measurements were performed analogously to the working experiments.

### 5.3. Atomic Force Microscopy

The AFM experiments were carried out using the direct surface adsorption method [8,15,35,36], similar to [8]. Briefly, muscovite mica sheets (SPI, West Chester, PA, USA) were used as AFM substrates. For AFM sample preparation, a freshly cleaved mica sheet was immersed into 800 µL of 0.1 μM aqueous HRP solution, which was incubated in the cell either within or far from the experimental setup, as described above. The AFM substrate was incubated in the HRP sample solution for 10 min at room temperature in a shaker at 600 rpm. After the incubation, each substrate was rinsed with 1 mL of ultrapure water, and then dried in air.

Mica surface with adsorbed HRP molecules was visualized by AFM. This method allows one to reliably measure the heights of single macromolecules with high (0.1 nm) resolution [3,4,5,8,15,16,17,25,27]. At the same time, lateral sizes of the resulting AFM images of the studied macromolecules can exceed their real sizes due to the effect of convolution of the probe and the studied objects [15,47]. For this reason, in our present study, only the height of the AFM images obtained was used for the determination of whether there is a change in the size of the mica-adsorbed HRP macromolecules. All AFM measurements were performed in tapping mode in air, employing a Titanium multimode atomic force microscope (NT-MDT, Zelenograd, Russia; the microscope pertains to the equipment of “Human Proteome” Core Facility of the Institute of Biomedical Chemistry, supported by the Ministry of Education and Science of Russian Federation, agreement 14.621.21.0017, unique project ID: RFMEFI62117X0017) equipped with NSG03 cantilevers (“TipsNano”, Zelenograd, Russia; 47 to 150 kHz resonant frequency, 0.35 to 6.1 N/m force constant). The total number of imaged objects in each sample was no less than 200, and the number of frames for each sample was no less than 10. Analogously to [36], the density of relative distribution of the imaged objects with height *ρ(h)* and the absolute number *N*_400_ of surface-adsorbed particles, normalized per 400 μm^2^ area, was calculated as described elsewhere [36].

Prior to the experiments with the HRP samples, preliminary experiments with the use of protein-free ultrapure water instead of protein solution were performed. No objects with a height greater than 0.5 nm were registered in the preliminary experiments.

AFM operation, obtaining AFM images, their treatment and exporting the obtained data in ASCII format were performed using a standard NOVA Px software (NT-MDT, Moscow, Zelenograd, Russia) supplied with the atomic force microscope. The number of the visualized particles in the obtained AFM images was calculated automatically using a specialized AFM data processing software developed in the Institute of Biomedical Chemistry (Rospatent registration no. 2010613458).

### 5.4. ATR-FTIR Measurements

To monitor the changes in the secondary structure of HRP, a VERTEX 70 V spectrometer (Bruker Scientific LLC, Billerica, MA, USA) was employed. The sample solution (8 μL) with a protein concentration of 10^−4^ M was placed into the measuring cuvette. Such a high concentration of the protein was chosen based on the sensitivity of the device employed. The data were presented in a standard form, according to the device software operation.

### 5.5. Spectrophotometric Measurements

HRP activity was estimated following the technique reported by Sanders et al. [48], employing ABTS as a reducing substrate—similar to our previous papers [8,35]. The rate of change in solution absorbance at 405 nm was monitored with an Agilent 8453 UV-visible spectrophotometer (Agilent Technologies Deutschland GmbH, Waldbronn, Germany). The HRP concentration in the quartz spectrophotometric cuvette was 10^−9^ M, while the concentrations of H_2_O_2_ and ABTS were 0.3 mM and 2.5 mM, respectively. Spectrum acquisition was started immediately after the addition of H_2_O_2_.

## Data Availability

Correspondence and requests for materials should be addressed to Y.D.I.

## Sample Availability

Samples of the compounds are not available from the authors.
